# Pleural empyema due to *Salmonella enterica* serovar Enteritidis in an immunocompetent elderly patient: a case report

**DOI:** 10.1099/jmmcr.0.005051

**Published:** 2016-08-30

**Authors:** Panagiota Xaplanteri, Stelios F. Assimakopoulos, Kostis Karachalios, Dimitrios Siagris, Alexandra Lekkou, Evangelos D. Anastassiou, Iris Spiliopoulou, Charalambos Gogos, Fevronia Kolonitsiou

**Affiliations:** ^1^​ Department of Microbiology, University General Hospital of Patras, Greece; ^2^​ Department of Internal Medicine, University General Hospital of Patras, Greece

**Keywords:** *Salmonella* Enteritidis, focal *Salmonella* Enteritidis infections, pleural empyema, dyspnea, ciprofloxacin

## Abstract

**Introduction:**

Pleural empyema as a focal infection due to *Salmonella enterica* serovar Enteritidis is rare and most commonly described among immunosuppressed patients or patients who suffer from sickle cell anaemia and lung malignancies.

**Case presentation:**

Here, we present an 81-year-old immunocompetent Greek woman with bacteraemia and pleural empyema due to *Salmonella* Enteritidis without any gastrointestinal symptoms.

**Conclusion:**

In our case, we suggest that patient’s pleural effusion secondary to heart failure was complicated by empyema and that focal intravascular infection was the cause of bacteraemia.

## Introduction

*Salmonellae* belong to the family Enterobacteriaceae and are motile Gram-negative facultative anaerobic bacilli. *Salmonella enterica* serovar Enteritidis mostly causes enterocolitis. Localised infections include gastroenteritis, osteomyelitis, nephritis, cholecystitis, endocarditis, meningitis and pneumonia. Pleural empyema is a rare localised infection due to bacteraemia from *Salmonella* Enteritidis and is usually associated with underlying immunodeficiency, sickle cell anaemia and lung cancer (Mandell *et al.*, 2010). Here, we present the case of an 81-year-old immunocompetent Greek female patient, who was diagnosed with pleural empyema due to *Salmonella* Enteritidis without any gastrointestinal symptoms, and we review the literature regarding bacteraemia and focal infections due to nontyphoidal *Salmonellae*.

## Case report

An 81-year-old Greek female patient with medical history significant for congestive heart failure, mild pulmonary hypertension, hypertension, atrial fibrillation and stroke in 2013 attended the Emergency Department of the University General Hospital of Patras due to dyspnoea and coughing for a week. She reported a single episode of fever 38 °C and chills 10 days before admission. No diarrhoea or symptoms related to gastrointestinal tract were reported. There was no former medical history of pulmonary disease or sickle cell anaemia.

The vital signs on admission were as follows: blood pressure of 137/60 mmHg, pulse rate of 81 beats/min, temperature of 37 °C and oxygen saturation of 78 % (FiO_2_ : 21 %). Physical examination revealed decreased breath sounds and dullness to percussion over the right lung base. The abdomen was soft on palpation without tenderness and there was lower extremities edema. The initial laboratory findings showed white blood cell count of 19.340 cells mm^−3^, with 83 % neutrophils, elevated C-reactive protein levels (34.3 U l^−1^, normal values <0.8 U l^−1^) and erythrocyte sedimentation rate of 118 mm in the first hour (normal values 0–20 mm). On admission, both renal and liver function parameters were elevated [blood urea 92 mg dl^−1^ (normal values 15–54 mg dl^−1^), creatinine 1.8 mg dl^−1^ (normal values 0.9–1.6 mg dl^−1^), aspartate aminotransferase 89 U l^−1^ (normal values 5–40 U l^−1^) and alanine aminotransferase 48 U l^−1^ (5–40)]. All these values dropped within normal range after treatment (blood urea 41 mg dl^−1^, creatinine 1.4 mg dl^−1^, aspartate aminotransferase 38 U l^−1^ and alanine aminotransferase 32 U l^−1^.

**Fig. 1.**
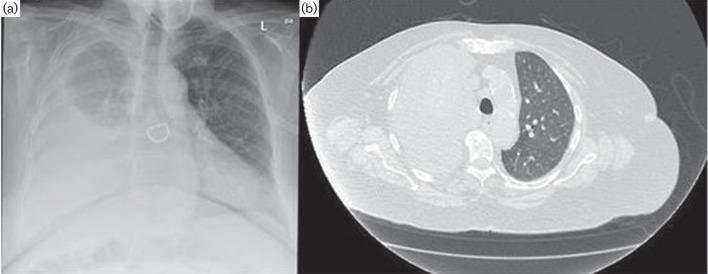
Patient’s chest X-ray (a) and CT scan (b) showing a large right-sided pleural effusion with lung atelectasis.

Posteroanterior chest X-ray (Fig. 1a) and computed tomography (CT) scan (Fig. 1b) showed a large right-sided pleural effusion partially encapsulated with lung atelectasis and a few in number up to 1.5 cm lymph nodes in the mediastinum. A diagnostic thoracocentesis was performed, which revealed a turbid exudative pleural fluid with the following parameters: pH: 7.5, specific weight: 1010, 1956 leukocytes/mm^3^ (64 % polymorphonuclear cells, 33 % lymphocytes and 3 % monocytes), 14 080 red blood cells/mm^3^, glucose 3.0 mg d l^−1^ (serum glucose 99 mg dl^−1^), lactate dehydrogenase 1769 U l^−1^ (serum lactate dehydrogenase 420 U l^−1^) and proteins 5.1 g dl^−1^ (serum proteins 7.8 g dl^−1^).

## Investigations

Further testing towards any possible associated immunodeficiency including human immunodeficiency virus (HIV) serology, serum globulin levels, full panel of serum tumour markers and glycosylated haemoglobin levels was unrevealing. Cytological pleural fluid analysis turned out to be negative for presence of malignant cells. In addition, no focal infection was identified in the gastrointestinal tract, heart and bones, according to results of abdominal CT scan, transthoracic echocardiography and bone scan, respectively. No stool culture was performed.

## Diagnosis

Samples of the pleural fluid were sent for culture and cytological analysis. On admission and during hospitalisation, blood cultures were also obtained. Blood and pleural fluid samples were inoculated into BAC/TAlert 3D (bioMerieux) blood aerobic culture bottles. Identification of the culpable microorganism was performed by Vitek® 2 Advanced Expert System (bioMerieux), whereas antibiotic susceptibility testing by the disk diffusion method for ceftriaxone and a gradient method (Etest, bioMerieux) for ciprofloxacin was according to the European Committee on Antimicrobial Susceptibility Testing guidelines (www.eucast.org). Serotyping was performed in all recovered isolates by polyvalent antisera (Statens Serum Institute). Both blood and pleural fluid cultures were positive for *S. enterica* serovar Enteritidis (AgO: 1, 9, 12).

## Treatment

Susceptibility testing showed susceptibility to ceftriaxone and ciprofloxacin (MIC=0.008 mg l^−1^). According to these results, the patient was treated with ciprofloxacin 400 mg every 12 h intravenously for a period of 10 days. Moreover, a chest tube was placed and 1.5 l of pleural fluid was drained.

## Outcome and follow-up

The patient was discharged on oral ciprofloxacin 500 mg every 12 h for 10 additional days after intravenous hospital therapy and recovered without complications. Follow-up blood cultures became negative after 7 days of treatment. No additional cultures of the pleural fluid after drainage were performed.

## Discussion

The clinical syndromes caused by *Salmonellae* in descending order of frequency include gastroenteritis, enteric fever, bacteraemia and localised infections. There is also the chronic carrier state that involves 0.2–0.6 % of the patients with nontyphoidal *Salmonella* infection. *Salmonella* Enteritidis typically causes gastroenteritis (Mandell *et al.*, 2010).

**Fig. 2.**
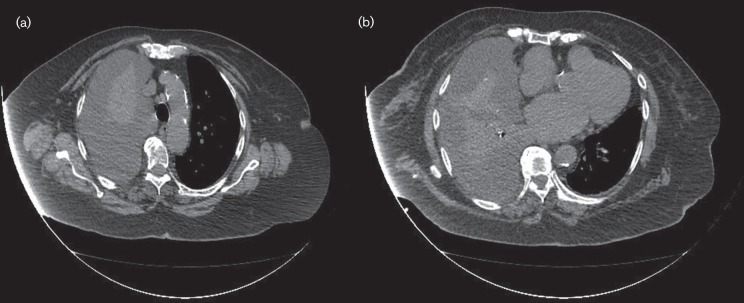
Patient’s CT scan showing the presence of calcified atherosclerotic plaques of the aortic arch (a) and descending aorta (b).

Bacteraemia develops in up to 8 % of patients with nontyphoidal *Salmonella* gastroenteritis and focal infection occurs mainly in infants, the elderly and patients with immunodeficiency. In contrast to children, focal infections due to primary bacteraemia are reported among adult patients and are related to increased mortality rates. In the elderly, *Salmonellae* invade atherosclerotic plaques and aneurysms during bacteraemia with a mortality rate up to 60 % (Mandell *et al.*, 2010). In our case, atherosclerosis was evident and based on the presence of calcified atherosclerotic plaques of the aortic arch and descending aorta, as shown in Fig. 2(a, b), respectively.

Primary *Salmonella* bacteraemia in combination with atherosclerotic plaques invasion seems a possible explanation as the mechanism of infection in our case. Our patient was elderly, with no symptoms from gastrointestinal tract prior to infection. Very few data are available on heart failure being a risk factor for *Salmonella* empyema. de Lope *et al.* reported a case of a 54-year-old patient suffering from mitral valvulopathy and left-sided ventricular failure who presented with *Salmonella* empyema (de Lope *et al.*, 2004). The annual incidence rate in patients above 50 years old with endovascular infection due to notyphoidal *Salmonellae* is 4.4 per 1 000 000 persons. Most of the patients reported in this group had a history of significant mycotic aneurysms or structural heart disease (Nielsen **et al.*,* 2006; Chen *et al.*, 2012; Ortiz *et al.*, 2014). Our patient had no history of valvulopathy or other structural heart disease, whereas no aneurysms were detected.

*Salmonella* Enteritidis extraintestinal focal infection is most commonly presented among immunosuppressed patients or patients with sickle cell anaemia (Table 1). Among humans with mutations in the genes encoding the IFN-γ and IL-12 receptor, or patients who undergo treatment with TNF inhibitors, bacteraemia due to nontyphoidal *Salmonella* is grave (Mandell *et al.*, 2010). Our patient had no medical history for sickle cell anaemia and there were no signs of defect in the IFN-γ and IL-12 receptor since there were no infections due to *Mycobacteria* or other intracellular bacteria during her childhood or afterwards. However, no tests were performed to examine IFN-γ and IL-12 receptor expression in our patient.

**Table 1.** Reported cases of pleural empyema caused by non typhoidal *Salmonella*

Author	Number of patients reported	Isolated microorganism	Underlying disease	Type of the infection
Buscaglia, 1978	1	*Salmonella* Newport	Splenic abscess	Pleural empyema
Chaturvedi *et al.*, 1978	1	*Salmonella* Newport	Sickle cell disease	Pleural empyema
Kate *et al.*, 1984	1	*Salmonella* typhimurium	Alveolar cell carcinoma	Pleural empyema
Santus *et al.*, 1984	1	*Salmonella* cholera suis	Metastasizing breast cancer	Pleural empyema
Ortiz *et al.*, 1991	1	*Salmonella* Enteritidis	Systemic lupus erythematosus	Pleural empyema
Gill *et al.*, 1996	1	*Salmonella* Enteritidis	Small cell bronchogenic carcinoma	Pleural empyema
Carel *et al.*, 1977	1	Nontyphoid *Salmonella*	Complication of a malignant pleural eﬀusion in an immunocompromised patient	Pleural empyema
Wolday *et al.*, 1997	1	*Salmonella* Enteritidis	HIV	Pleural empyema
Roguin *et al.*, 1999	1	*Salmonella* Mendoza	Myelodysplastic syndrome	Pleural empyema
Ramanathan *et al.*, 2000	2	*Salmonella* Senftenberg	One patient with diabetes mellitus and the other one with gallbladder carcinoma	Pleural empyema
Rim *et al.*, 2000	1	*Salmonella* Group B	Diabetes mellitus	Pleural empyema
de Lope *et al.*, 2004		*Salmonella* Enteritidis	Age over 50 years old	Pleural empyema
Crum *et al.*, 2005	1	Nontyphoid *Salmonella*	Immunosuppression	Pleural empyema
Takiguchi *et al.*, 2008	1	*Salmonella* Livingstone	Tuberculosis	Chronic empyema
Yang *et al.*, 2008	1	Nontyphoid *Salmonella*	A 26-year-old male patient, immunocompetent	Pleural empyema
Kam *et al.*, 2012	1	Group D *Salmonella*	Underlying pulmonary pathology, secondary to an extensive smoking history	Pleural empyema
Pathmanathan *et al.*, 2015	1	*Salmonella* typhimurium	Mild neutropenia (1.25×109 l^−1^)	Pleural empyema

*Salmonella* is an intracellular pathogen, and it is well known that the final clearance of the infection depends on cellular immunity. Accordingly, diseases predisposing to extraintestinal *Salmonella* Enteritidis infection are AIDS, inflammatory bowel disease, lung and blood malignances (like leukaemia and lymphomas), diabetes mellitus, profound alcohol consumption, prolonged corticosteroid therapy and antineoplastic treatments (Table 1) (Rim *et al.*, 2000; Mandell *et al.*, 2010). It seems that 33.3 % of the HIV-infected patients diagnosed with *Salmonella* bacteraemia had focal lung infections (Casado *et al.*, 1997; Fernandez-Guerrero *et al.*, 1997; Persson *et al.*, 2000; Samonis *et al.*, 2003). Our patient proved to be negative for HIV serotypes 1 and 2. Moreover, blood glucose and glycosylated haemoglobin levels were normal and she did not prove to suffer from any malignancy. She was not consuming alcohol regularly and was not under corticosteroid or antineoplastic therapy.

The incidence of focal pulmonary infection due to *Salmonella* Enteritidis bacteraemia is very low and is regarded to be less than 1 % (Mandell *et al.*, 2010). *Salmonellae* in the blood stream seem to have a tropism for abnormal tissues like malignant tumours, bone infarcts and aneurysms, whereas focal intravascular infection accounts for 25 % of patients over 50 years old (Khan *et al.*, 1983; Samonis *et al.*, 2003; Mandell *et al.*, 2010). Despite pneumonia lung abscess, rarely pleural empyema, most commonly in patients with immunosuppression or underlying lung disease, has been described (Table 1) (Khan *et al.*, 1983; Mandell *et al.*, 2010; Kam *et al.*, 2012). As mentioned above, our patient proved to be immunocompetent and did not suffer from lung disease.

*Salmonella* Enteritidis is reported to be a rare pathogen causing pleural empyema infection. Up to 2015, at about 17 cases of *S. enterica* empyema without pulmonary implication were published in Korea, USA, Spain, Japan, Italy, Ethiopia, Israel and India, as shown in Table 1 (Carel *et al.*, 1977; Buscaglia, 1978; Chaturvedi *et al.*, 1978; Kate *et al.*, 1984; Santus *et al.*, 1984; Ortiz *et al.*, 1991; Gill *et al.*, 1996; Wolday *et al.*, 1997; Roguin *et al.*, 1999; Ramanathan *et al.*, 2000; Rim *et al.*, 2000; de Lope *et al.*, 2004; Takiguchi *et al.*, 2008; Yang *et al.*, 2008; Kam *et al.*, 2012; Crum *et al.*, 2005; Pathmanathan *et al.*, 2015). A case reported by Yang **et al.** (2008) refers to a 26-year-old immunocompetent male patient presented with nontyphoid *Salmonella* pleural empyema with previous history of pneumonia. To our knowledge, in Greece, there is only one report of *S. enterica* serotype Enteritidis pneumonia regarding a 72-year-old male patient with lung cancer in the island of Crete, in 2003. This was the first case of immunodeficient patient with *Salmonella* Enteritidis bacteraemia and empyema in Greece (Samonis *et al.*, 2003). Our patient is the second case worldwide (de Lope **et al.*,* 2004) and the first in Greece, presented with pleural empyema caused by *Salmonella* Enteritidis in elderly patient with no severe underlying disease or pneumonia.

It has been reported that nontyphoid *Salmonellae* rest dormant in the reticuloendothelial system and blood spread is a consequence of reactivation. Moreover, due to low bacterial load, blood cultures are often negative. It has also been reported that *Salmonellae* seed atheromatic plaques and this can be the source of bacteraemia (Mandell *et al.*, 2010; Pathmanathan *et al.*, 2015). Our patient presented with lower extremities edema, dyspnoea and right-sided pleural effusion was already under treatment for congestive heart failure. These clinical findings support the suspicion that in this case *Salmonella* bacteraemia complicated a preexisting pleural effusion secondary to heart failure, as already reported (de Lope *et al.*, 2004). In elderly patients with atherosclerosis and congestive heart failure, *Salmonella* bacteraemia must be considered a possible mechanism of pleural effusion dissemination. Even so, the incident of pleural empyema due to *Salmonella* Enteritidis is extremely low.
